# Accumulation of Palmitoylcarnitine and Its Effect on Pro‐Inflammatory Pathways and Calcium Influx in Prostate Cancer

**DOI:** 10.1002/pros.23222

**Published:** 2016-07-12

**Authors:** Ala'a Al‐Bakheit, Maria Traka, Shikha Saha, Richard Mithen, Antonietta Melchini

**Affiliations:** ^1^ Department of Nutrition and Food SciencesAl‐Balqa’ Applied UniversityAl‐SaltJordan; ^2^ Food and Health ProgrammeInstitute of Food ResearchNorwichUnited Kingdom

**Keywords:** acylcarnitines, metabolic disruption, interleukine‐6 pathway, intracellular calcium signaling, dihydrotestosterone

## Abstract

**BACKGROUND:**

Acylcarnitines are intermediates of fatty acid oxidation and accumulate as a consequence of the metabolic dysfunction resulting from the insufficient integration between β‐oxidation and the tricarboxylic acid (TCA) cycle. The aim of this study was to investigate whether acylcarnitines accumulate in prostate cancer tissue, and whether their biological actions could be similar to those of dihydrotestosterone (DHT), a structurally related compound associated with cancer development.

**METHODS:**

Levels of palmitoylcarnitine (palcar), a C16:00 acylcarnitine, were measured in prostate tissue using LC‐MS/MS. The effect of palcar on inflammatory cytokines and calcium (Ca^2+^) influx was investigated in in vitro models of prostate cancer.

**RESULTS:**

We observed a significantly higher level of palcar in prostate cancerous tissue compared to benign tissue. High levels of palcar have been associated with increased gene expression and secretion of the pro‐inflammatory cytokine IL‐6 in cancerous PC3 cells, compared to normal PNT1A cells. Furthermore, we found that high levels of palcar induced a rapid Ca^2+^ influx in PC3 cells, but not in DU145, BPH‐1, or PNT1A cells. This pattern of Ca^2+^ influx was also observed in response to DHT. Through the use of whole genome arrays we demonstrated that PNT1A cells exposed to palcar or DHT have a similar biological response.

**CONCLUSIONS:**

This study suggests that palcar might act as a potential mediator for prostate cancer progression through its effect on (i) pro‐inflammatory pathways, (ii) Ca^2+^ influx, and (iii) DHT‐like effects. Further studies need to be undertaken to explore whether this class of compounds has different biological functions at physiological and pathological levels. *Prostate 76:1326–1337, 2016*. © 2016 The Authors. *The Prostate* published by Wiley Periodicals, Inc.

## INTRODUCTION

The aetiology of prostate cancer is multi‐factorial and includes many metabolic dysregulations 1. Prostate carcinogenesis is characterized by a unique reprogramming of metabolic processes, such as lipogenesis and glycolysis, in order to provide sufficient energy to support cancer cell growth 1, 2, 3, 4. Induction of these metabolic processes is accompanied with an increase in the levels of reactive oxygen species (ROS), which can negatively affect key enzymatic activities involved in the tricarboxylic acid (TCA) cycle function 5. A less efficient TCA cycle results in a poor co‐ordination with β‐oxidation leading to the accumulation of intermediates of fatty acid oxidation, such as acylcarnitines 6.

Accumulation of acylcarnitines has been observed in chronic conditions, such as diabetes mellitus (DM) type 2 and obesity, and also has been positively correlated with mitochondrial dysfunction and markers of metabolic syndrome 7, 8. There is an increasing body of evidence suggesting that accumulation of acylcarnitines may provide a possible biomarker for insulin resistance associated with DM type 2 and obesity 6, 7, as well as cardiovascular diseases (CVD) and inflammatory conditions associated with metabolic syndrome 7. Recent metabolomic studies have reported an increased level of carnitines and acylcarnitines in tissue, serum, and urines from cancer patients compared to healthy controls 9, 10, 11, 12. Giskeodegard and colleagues have found increased levels of acylcarnitines in plasma samples from prostate cancer patients compared with control patients with benign prostatic hyperplasia (BPH) 13.

Palmitoylcarnitine (palcar), C16:00 acylcarnitine is formed from palmitic acid (PA) that represents approximately 80% of the total fatty acids synthesized in the cell 14. Numerous studies have been undertaken to quantify palcar levels in animal and human tissue (Table I) 7, 15, 16, 17, 18, 19, 20. However, the levels of palcar and the biological consequences of its accumulation have not been investigated in prostate cancer.

**Table I:** Levels of Palcar as Reported in the Literature

Palcar			
Healthy levels	Unhealthy levels	Biological material	References
0.12 nmol/µL	0.57 nmol/µL	Serum (human)	12
0.25 μM	1.02 μmol/L (VLCAD)^a^ 1.55 μmol/L (GA‐II)^b^	Plasma (human)	14
1.70 μM	n/a	Cord blood samples (human)	15
2.16 μM	n/a	Heel‐prick blood samples (human)	15
0.04 ± 0.02 μM	0.07 ± 0.02 μM (obese); 0.06 ± 0.03 μM (diabetic)	Plasma (human)	16
0.352 ± 0.148 μM	0.404 ± 0.220 μmole/L (chronic fatigue syndrome)	Plasma (human)	17
0.92 μM	n/a	Blood spot (human)	18
∼12 μM	n/a	Skeletal muscle (human)	18
∼17 μM	∼19 μM (high fat diet)	Adipose tissue (rat)	7
∼6 μM	∼12 μM (high fat diet)	Muscles (rat)	7
n/a	60 μM (ischemic heart)	Heart (rabbit)	19

^a^ VLCAD: very long chain acyl‐CoA dehydrogenase.

^b^ GA‐II: glutaric acidemia type II.

This study aimed to determine whether palcar levels are different between non‐cancerous and cancerous prostate tissue, and then explore its biological effects in in vitro models of prostate cancer. Pro‐inflammatory pathways have been associated with the development of prostate cancer 21, 22, and acylcarnitines have been found to exacerbate these pathways 7, 23. The current study has explored the effects of palcar on the pro‐inflammatory cytokine IL‐6 in prostate cells. Calcium (Ca^2+^) is an important secondary ion that is involved in many signaling pathways, some of which are related to inflammatory responses such as IL‐6 production 24. Acylcarnitines have been shown to alter membrane proteins that act as ion channels and pumps including Ca^2+^ pumps 25, 26, 27. The alteration of membrane permeability induced by acylcarnitines leads to a change of ion concentrations that may be involved in inflammatory signaling pathways 28. It has been reported that palcar is able to induce Ca^2+^ influx in endothelial cells 25, 29 which was also observed in response to dihydrotestosterone (DHT) 30, a hormone linked to prostate cancer progression. This study has compared the effect of palcar on Ca^2+^ influx to that induced by the structurally related compound, DHT, in prostate cells. Furthermore, a global gene expression analysis has been carried out to determine whether palcar has androgen‐like biological activity.

## MATERIALS AND METHODS

### Chemical Reagents and Solutions

Internal standard of d3‐palmitoyl‐carnitine (D3‐C16:0) and d3‐octanoylcarnitine (L‐carnitine:HCL, O‐Octanoyl, N‐Methyl‐D3, 98%, Cat # DLM‐755‐0) were purchased from Cambridge Isotope Laboratories Inc., Tewksbury, MA). Stock solutions were prepared in DMSO and further diluted using acetonitrile and methanol (3:1). Palcar (Cat # P4509, Sigma–Aldrich, Dorset, UK), DHT (Cat # 31573‐100MG, Sigma–Aldrich) and FURA‐2 acetoxymethyl ester (FURA‐2AM) (Cat # F0888, Sigma–Aldrich) stock solutions were all prepared in DMSO. Histamine (Cat # H7125‐1G, Sigma–Aldrich) was prepared in de‐ionized water. All stock solutions were diluted to the required final concentrations using media supplemented with 10% foetal bovine serum (FBS).

### Prostate Tissues and Sample Preparation

Tissue levels of palcar were quantified from non‐cancerous (n = 10) and cancerous (n = 10) prostate tissue samples. Frozen tissue samples were obtained from The Norwich Biorepository (Norwich and Norfolk University Hospital, UK). Ethical approval was gained prior to commencing the study from the Faculty Research Ethics Committee of the University of East Anglia (Norwich, UK) (reference: 2012/2013–10HT; 2012/2013–37). Upon receipt, tissues were transformed into a fine powder using a tissue BioPulverizer (Cat # 59012N, Bio Spec Products Inc., Bartlesville, OK) under liquid nitrogen and stored at −80°C until required for analysis. Palcar tissue levels were measured by liquid chromatography‐mass spectrometry (LC‐MS/MS) following a published method 31 with slight modifications. Frozen tissue powder (∼20 mg) were mixed with 50 μl of freshly prepared 1 M potassium phosphate monobasic (KH_2_PO_4_) solution and 10 μl of 100 ng/ml of each of internal standards solutions; d3‐Octanoylcarnitine (D3‐C8:0) and d3‐palmitoyl‐carnitine (D3‐C16:0). Then, 500 μl of freshly prepared extraction solution of 3:1 acetonitrile:methanol (v/v) was added. The resulting mixture was vortexed for 30 sec and further homogenized with a mechanical grinder for 30 sec, then another 500 μl of the freshly prepared extraction solution was added to each sample and homogenized for an additional 30 sec and vortexed again. After centrifugation at 13,000 rpm for 5 min at 4°C, 2 μl of the supernatants were automatically injected on Waters Acquity UPLC BEH C8 column (1.7 μm particle size, 50 × 21 mm) using mobile phase with volatile ion‐pairing reagent consisting of 10 mM heptafluorobutyric acid and 10 mM ammonium acetate in water (mobile phase A) and in methanol (mobile phase B), at a flow rate of 0.4 ml/min with linear gradient. Agilent 6490 Mass spectrometry analysis was performed with the use of electrospray ionization in positive ion polarity by MRM mode with gas and sheath gas temperatures 200 and 400°C, respectively, gas flow 16 L/min, Nebulizer pressure 60 psi, sheath gas flow 12 L/min and capillary voltage 3,500 V. The mass (m/z) of the selected precursor (parent ion) 400 and product ion 85 m/z were selected for palcar monitoring by MRM mode. Palcar tissue levels were determined using a standard curve that was prepared using serial dilutions of the synthetic palcar stock solution.

### Cell Culture

Human normal prostate epithelial (PNT1A) (Cat # 95012614), human prostate adenocarcinoma PC3 (Cat # 90112714) and LnCap clone FGC (Cat # 89110211) cell lines were obtained from the European Collection of Cell Culture. Human benign prostatic hyperplasia epithelial (BPH‐1) cell line was obtained from the German collection of microorganism and cell cultures (No. ACC 143, DSMZ, Germany). Human prostate adenocarcinoma DU145 cell line was purchased from the American Type Culture Collection (Cat # HTB‐81). PNT1A, LnCaP and BPH‐1 cells were routinely cultured in RPMI media (Cat # E15‐885, PAA cell‐culture Company, Little Chalfont, UK) supplemented with 10% FBS (Sigma–Aldrich). DU145 and PC3 cells were cultured in EMEM (Cat # M2279, Sigma–Aldrich Co.) and HAMS (Cat # BE02‐014F, Lonza Verviers sprl, UK) media, respectively, both supplemented with 10% FBS. All cell lines were grown to 70–80% confluent at 37°C in a humidified atmosphere of 5% CO_2_.

### WST‐1 Viability Assay

Cell viability in response to palcar was determined using WST‐1 viability assay. PNT1A and PC3 cells were seeded in 96‐well culture plates in a final volume of 100 μl/well culture medium and cultured in a humidified atmosphere (37°C, 5% CO_2_). Cells were allowed to adhere to the plate surface for 36 hr before being treated with palcar (0–100 μM) or vehicle control (DMSO) for 24 hr. Each dose of palcar was tested six times. WST‐1 reagent (10 μl) was added to each well and incubated for 30 min in a humidified atmosphere (37°C, 5% CO_2_). Quantification of the formazan dye produced by metabolically active cells was performed by a scanning multiwell spectrophotometer, measuring absorbance at 450 nm by microplate ELISA reader (ELx808, Ultra Microplate Reader; BIO‐TEK Instruments, Inc., Winooski, VT).

### Measuring IL‐6 Secretion in Response to Palcar

PNT1A, PC3, and LnCaP cells were grown to 70–80% confluence before being treated with palcar (0–100 μM) or vehicle control (DMSO) for 24 hr in a humidified atmosphere (37°C, 5% CO_2_). Supernatants were then collected and appropriately stored at −20°C until analysis. IL‐6 levels were quantified by using a commercially available ELISA kit (Cat # D6050, R&D Systems, UK) following manufacturer's instructions.

### Measuring IL‐6 Gene Expression

PNT1A and PC3 cells were grown to 70–80% confluence before treatment with palcar (0–100 μM) or vehicle control (DMSO). The treatment was carried out for 24 hr in a humidified atmosphere (37°C, 5% CO_2_). Total RNA was extracted using RNeasy mini kit (Cat # 74104, Qiagen, UK) following the manufacturer's procedure. The quantity and quality of RNA was determined using RNA Nanodrop 1000 (Life Technologies, Loughborough, UK). The ratio of 260/280 was 1.9–2.1 for all samples. IL‐6 gene expression was measured by real time RT‐PCR performed using an Applied Biosystems OneStep Plus real time RT‐PCR system on an optical 96‐well plate in a total volume of 20 μl/well, consisting of TaqMan 1‐step RT‐PCR master mix reagent kit (Cat # 4392938, Life technologies, Invitrogen, UK), 20 ng total RNA, and IL‐6 primers and probes: IL‐6 forward sequence [5′‐CTCTTCAGAACGAATTGACAAACAAAT‐3, 100 μM, reverse sequence 5′‐ATGTTACTCTTGTTACATGTCTTCTTTCTC‐3, 100 μM and probe 5′‐TACATCCTCGACGGCATCTCAGCCC‐3′, 100 μM]. All were designed and purchased from Eurofins (UK). Reverse transcription was performed for 30 min at 48°C, amplification Taq activation for 10 min at 95°C, followed by 40 PCR cycles of denaturation at 95°C for 15 sec and annealing/extension at 60°C for 1 min. Reactions were carried out in triplicate and were normalized against an endogenous housekeeping gene, 18 S ribosomal RNA. The amount of mRNA and thus gene expression was quantified based on a standard curve method.

### Measuring Ca^2+^ Influx

Human prostate cells (PNT1A, BPH‐1, DU145, PC3) were cultured to 70–80% confluence, and then they were harvested by using trypsin. Cell suspension in 10% FBS‐supplemented media was centrifuged at 1,500 rpm for 3 min at room temperature. Cell pellet was re‐suspended in 10% FBS‐supplemented media and then incubated for 1 hr at 37°C and 5% CO_2_. Cells were counted and FURA‐2AM at the final concentration of 250 nM was added to the cell suspension (1 × 10^6^ cells/ml) for 30 min at 37°C and 5% CO_2_. After washing, cells (100 μl) were seeded in a black 96‐well plate with clear bottom and were immediately treated with vehicle control, histamine (0–20 μM), palcar (0–50 μM) or DHT (0–1 μM). The kinetic of fluorescence was measured using a fluorescence plate reader at 510 nm (excitation) and at both 340 and 380 nm (emission) every 10 sec intervals for 5 min. Ca^2+^ influx has been expressed in terms of fluorescence ratio.

### Microarray Analysis

PNT1A cells were cultured to 70–80% confluence, and then they were treated with DMSO, palcar (0–5 μM) and DHT (10 nM) for 8 hr at 37°C and 5% CO_2_. RNA was extracted using RNeasy Mini kit as described in the manufacturer's instruction. RNA was quantified by using the Nanodrop 1000 spectrophotometer (Life Technologies). RNA quality was analysed by using Agilent Bioanalyser. Gene expression profiling was performed using Affymetrix GeneChip Human Exon 1.0ST Array (Affymetrix Sana Clara, CA) at Nottingham Arabidopsis Stock Centre (Nottingham, UK) following the Affymetrix protocols. Data were analysed using R/Bioconductor 32 and the aroma.affymetrix package 33. Data were robust multiple average (RMA) background‐corrected and quantile normalized. To obtain the gene‐level summaries, linear probe level models were applied to the data. For annotation, the current custom CDF file available at the aroma.affymetrix Web site containing the core probe sets (18708 transcript clusters; 284258 probe sets) was used. Subsequent statistical data analysis to identify differentially expressed genes was performed using limma 34. Genes were identified as differentially expressed at different Benjamini and Hochberg adjusted *P* values. To identify pathways that were the most over‐presented in the lists of differentially expressed genes, functional analyses using the Database for Annotation, Visualization and Integrated Discovery v6.7 (DAVID; http://david.abcc.ncifcrf.gov/) was used 35.

### Statistical Analyses

Statistical analysis of palcar tissue levels was performed by using unpaired *t*‐test. Data obtained from IL‐6 gene expression and secretion analyses were expressed as mean ± standard deviation (SD). Outlier removal and check for normality of residuals were performed before statistical comparisons of the results, made using one‐way analysis of variance (ANOVA) followed by Bonferroni's multiple comparison post‐test. Statistical analysis of Ca^2+^ influx data was performed with mixed model test using Genstat software.

## RESULTS

### Levels of Palcar in Prostate Non‐Cancerous and Cancerous Tissues

**Figure 1:**
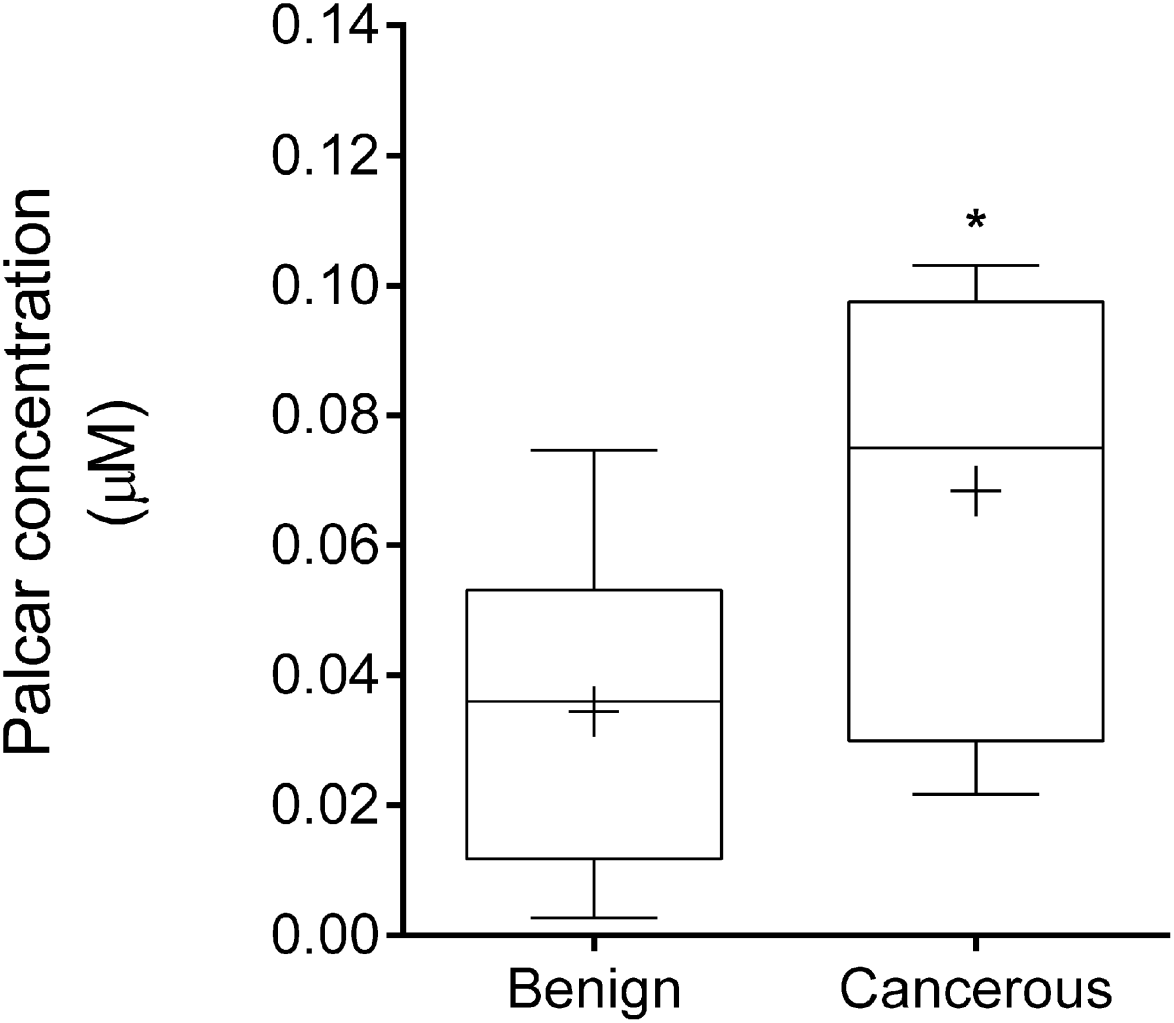
Concentration of palcar in human prostate tissue. Palcar concentration (μM) was measured by LC/MS‐MS in benign (n = 10) and cancerous (n = 10) tissue samples. Mean values are shown as “+.” Statistical analysis was performed with unpaired *t*‐test (*P* = 0.015).

LC‐MS/MS analysis revealed a significantly higher level of palcar in the prostate cancerous tissue (0.068 ± 0.03 μM) compared to the non‐cancerous tissue (0.034 ± 0.02 μM) (*P* = 0.015, Fig. 1). As expected, individual differences in the quantified levels of palcar were observed in both non‐cancerous and cancerous prostate tissue.

### Effects of Palcar on the Pro‐Inflammatory Cytokine IL‐6

**Figure 2:**
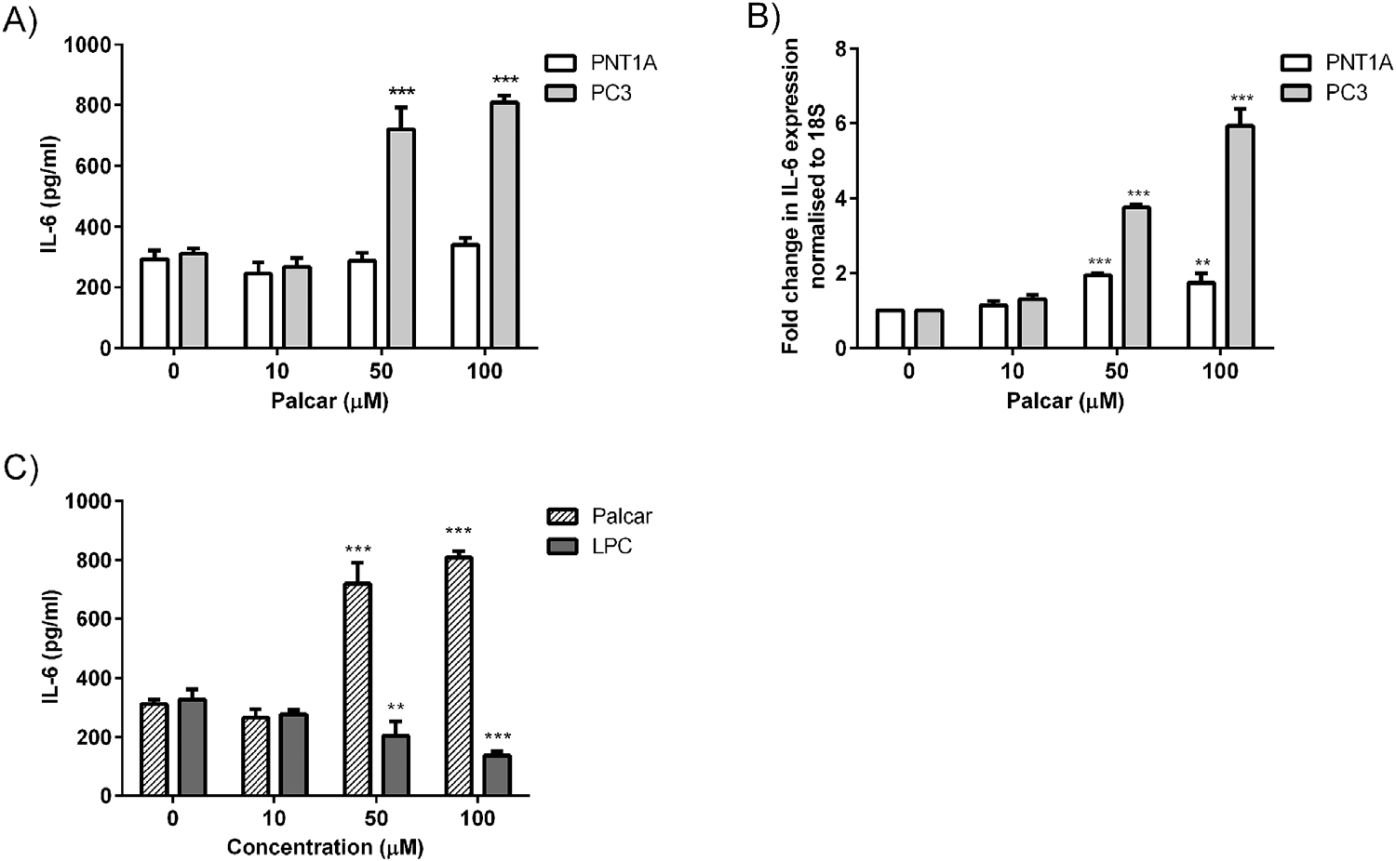
Effect of palcar on the pro‐inflammatory cytokine IL‐6. (**A**) IL‐6 secretion in PNT1A and PC3 cells in response to palcar as measured in culture medium using human IL‐6 ELISA kit. (**B**) IL‐6 gene expression in PNT1A and PC3 cells in response to palcar. IL‐6 gene expression was quantified using real time RT‐PCR and housekeeping 18 S gene was used for normalisation. (**C**) IL‐6 secretion in response to palcar and LPC in PC3 cells. Statistical analysis was performed using a one way ANOVA followed by Bonferroni's multiple comparison post‐test (***P < 0.001, **P ≤ 0.01, *P < 0.05 vs. untreated cells).

To assess the pro‐inflammatory effects of palcar, IL‐6 secretion was measured in response to palcar treatment (0–100 μM) for 24 hr. Palcar induced a significant increase in IL‐6 secretion in androgen‐independent cancerous PC3 cells only at concentrations as high as 50 μM (*P* ≤ 0.01). Palcar treatment was not associated with any increase in IL‐6 secretion in normal PNT1A cells (Fig. 2A). Basal IL‐6 level was undetectable in androgen‐dependent cancerous LnCaP cells, and no effect of palcar was observed at any of the concentrations tested (0–100 μM) (data not shown). To investigate whether the effect of high concentrations of palcar on IL‐6 secretion was due to change at the gene level, IL‐6 gene expression was measured. Palcar (50–100 μM) induced a significant increase in IL‐6 gene expression in both PNT1A and PC3 cells that was more pronounced in the cancerous PC3 cell line (≤sixfold increase) (*P* ≤ 0.001) (Fig. 2B). The effect of palcar on inducing IL‐6 secretion in PC3 cells was compared with lysophosphatidycholine (LPC), a lipid related compound derived from phosphatidylcholine and exhibiting amphiphilic properties similar to that of palcar. Figure 2C shows the levels of both LPC and palcar‐induced IL‐6 secretion (pg/ml). After 24 hr exposure, LPC significantly reduced the secretion of IL‐6 at concentrations as high as 50 μM in PC3 cells (*P* < 0.001). The observed effect of LPC was opposite to that observed in response to palcar.

### Cell Viability in Response to Palcar in PNT1A and PC3 Cells

**Figure 3:**
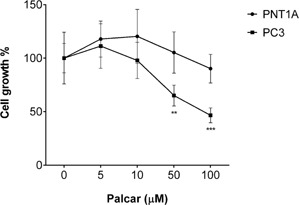
Effect of palcar on cell growth. Viability assay in PNT1A and PC3 cells in response to palcar treatment (0–100 μM) for 24 hr. Results represent means ± SD of six biological replicates (*P ≤ 0.05, **P ≤ 0.01; one way ANOVA followed by Tukey's multiple comparison post‐test).

Results obtained from the WST‐1 assay show that treatment with palcar (0–100 μM) was not associated with any toxic effect in PNT1A cells, whereas high concentrations of palcar (>50 μM) induced a significant decrease in PC3 cell viability (*P* ≤ 0.01) (Fig. 3).

### Effects of Palcar on Ca^2+^ Influx

**Figure 4:**
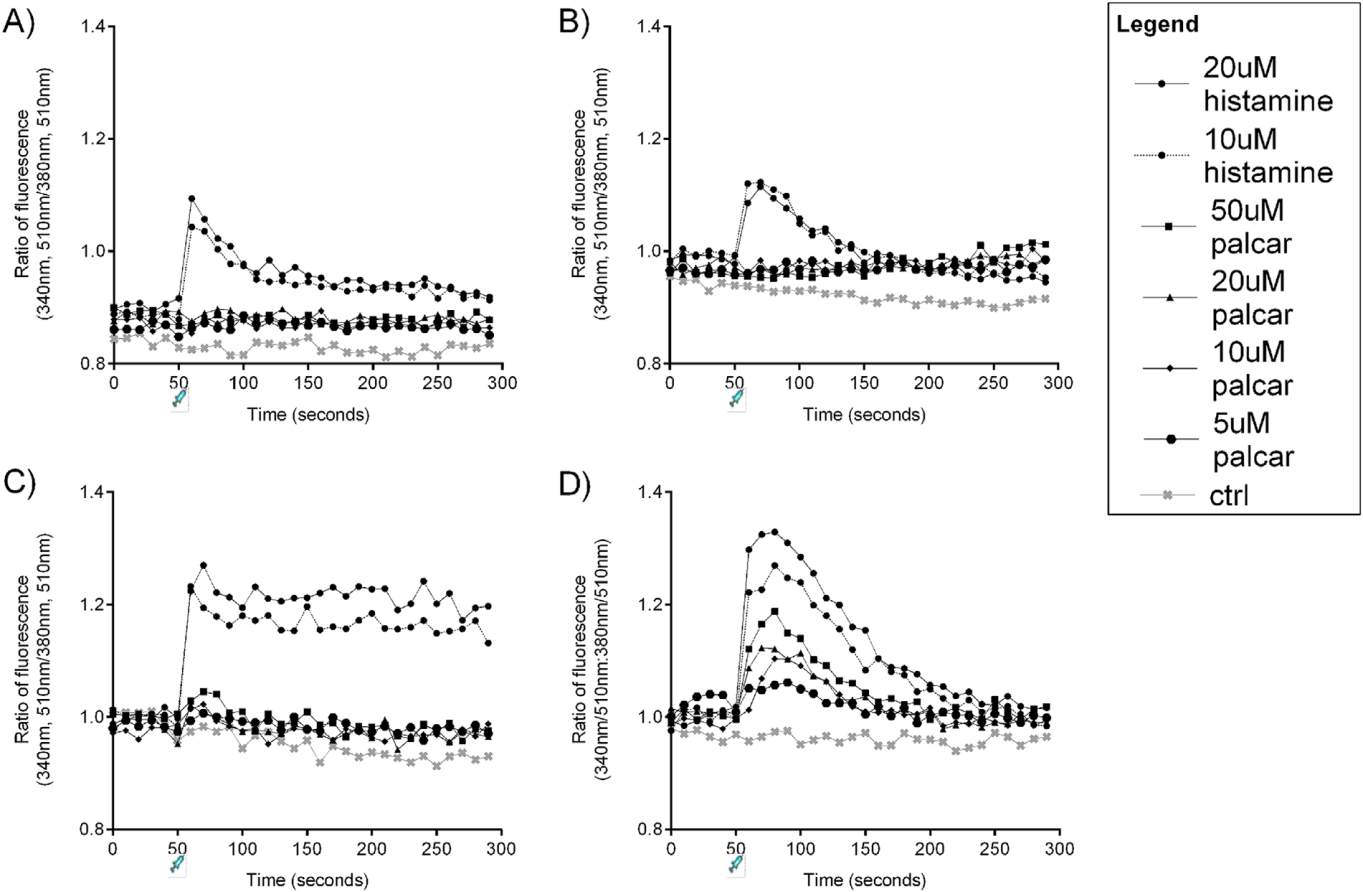
Effect of palcar on Ca^2+^ influx. FURA‐loaded PNT1A (**A**), BPH‐1 (**B**), DU145 (**C**), and PC3 (**D**) cells were injected with DMSO (control), histamine (10–20 μM) or palcar (5–50 μM) 50 sec after starting Ca^2+^ measurement. The ratio of fluorescence emission (510 nm), excited at both 340 nm and 380 nm, indicated the [Ca^2+^]i. Data represent mean values of three independent experiments. The statistical analysis was performed with mixed effect model test using the Genstat software. Ratio of fluorescence after histamine injection was significantly different from control in all four cell lines (*P* ≤ 0.01). Ratio of fluorescence after palcar injection was not significantly different from the control in PNT1A (*P* = 0.110), BPH‐1 (*P* = 0.521), and DU145 cells (*P* = 0.110). In PC3 cells, palcar‐induced Ca^2+^ influx was significantly different from the control (*P* = 0.011), but there was no significant difference within the different concentrations of palcar (*P* = 0.839).

To investigate the effect of palcar on Ca^2+^ influx, non‐cancerous and cancerous prostate cell lines (PNT1A, BPH‐1, DU145, and PC3) were exposed to a range of palcar concentrations (0–50 μM), and histamine was used as a positive control. Before exposing the cells to histamine or palcar no change in the fluorescence ratio was observed (Fig. 4). Treatment with either 10 or 20 μM histamine caused a rapid and immediate increase in Ca^2+^ influx and this was in a dose response manner (PNT1A and BPH‐1, *P* ≤ 0.001; DU145, *P* = 0.003; PC3, *P* = 0.01). Treatment with palcar (5–50 μM) was not able to induce Ca^2+^ influx in PNT1A and BPH‐1 cell lines (Fig. 4A and B), which was also observed in the cancerous DU145 cells (Fig. 4C). However, palcar was able to induce Ca^2+^ influx in a dose response manner in PC3 cells (*P* = 0.011) (Fig. 4D). The maximum influx in PC3 cells was observed after 80 sec of palcar addition and lasted for approximately 2 min.

### Effects of DHT on Ca^2+^ Influx

**Figure 5:**
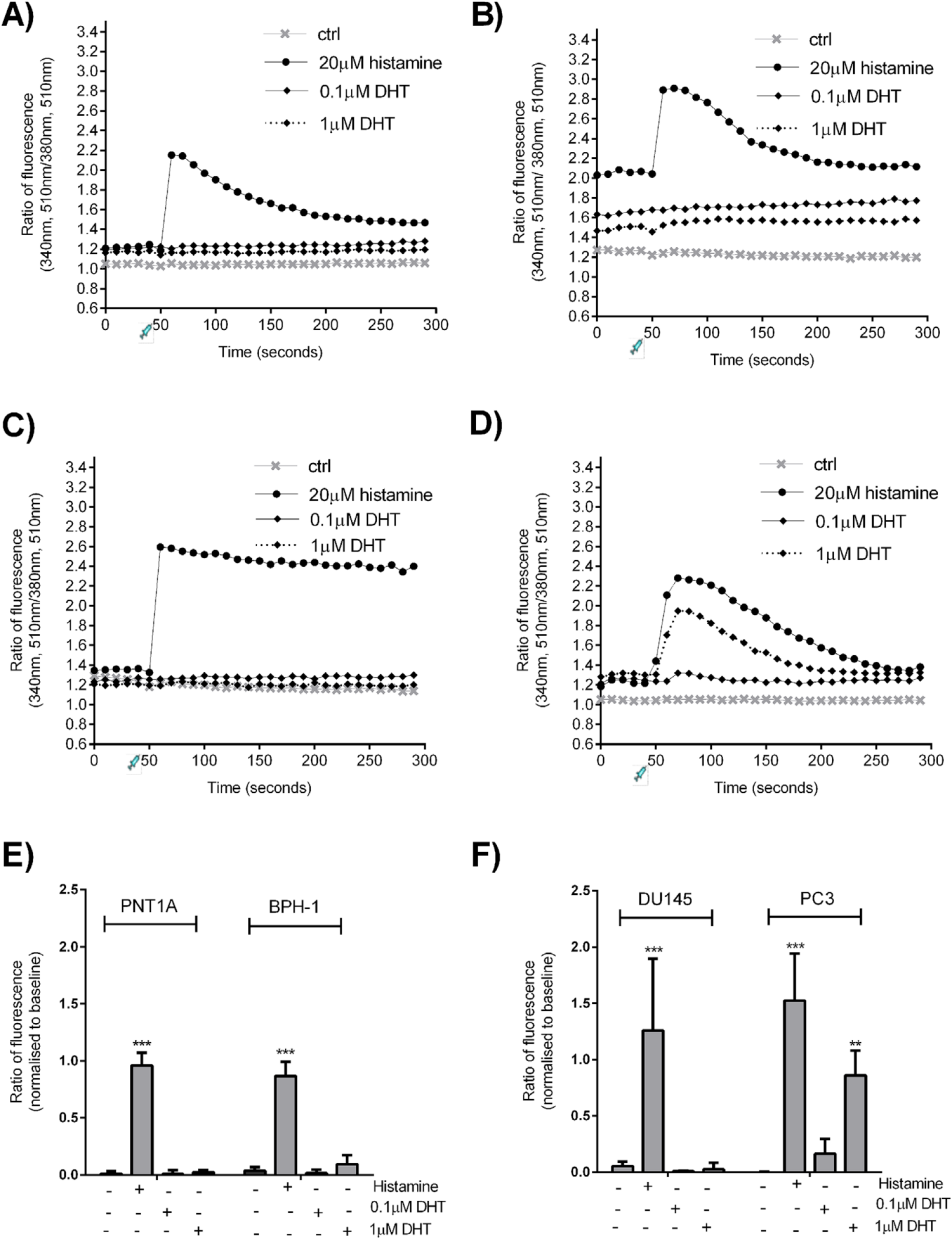
Effect of DHT on Ca^2+^ influx. FURA‐loaded PNT1A (**A**), BPH‐1 (**B**), DU145 (**C**), and PC3 (**D**) cells were injected with DMSO (control), 20 μM histamine or DHT (0.1‐1μM) 50 sec after starting Ca^2+^ measurement. The ratio of fluorescence emission (510 nm), excited at both 340 nm and 380 nm, indicated the [Ca^2+^]i. (**E**,**F**) The maximum fluorescence ratio was normalized to the baseline level at 50 sec of each sample injection. Data represent means ± SD of three independent experiments. Statistical analysis was performed using two ways ANOVA followed by Bonferroni's post‐test (*P ≤ 0.05, **P ≤ 0.01, ***P ≤ 0.001 vs. untreated cells).

Since both palcar and DHT are lipid related compounds, the effects of palcar on Ca^2+^ influx was compared to those induced by DHT in order to investigate whether structural similarity could explain a similar biological profile. Prostate cells (PNT1A, BPH‐1, DU145, and PC3) were exposed to DHT (0.1 and 1 μM), and histamine (20 μM) as a positive control. As expected, histamine was able to significantly increase fluorescence ratio in all cell lines (*P* ≤ 0.001). Treatment with either 0.1 or 1 μM DHT was not associated with an increase in Ca^2+^ influx in PNT1A, BPH‐1 and DU145 cells (Fig. 5A–C). A rapid increase in Ca^2+^ influx was observed in PC3 cells only at the concentration of 1μM DHT (*P* ≤ 0.01) (Fig. 5D). The maximum fluorescence ratio was normalized to the baseline level at 50 sec of each sample injection (Fig. 5E and F).

### Effects of Palcar and DHT on Gene Expression in Non‐Cancerous PNT1A Cells

**Figure 6:**
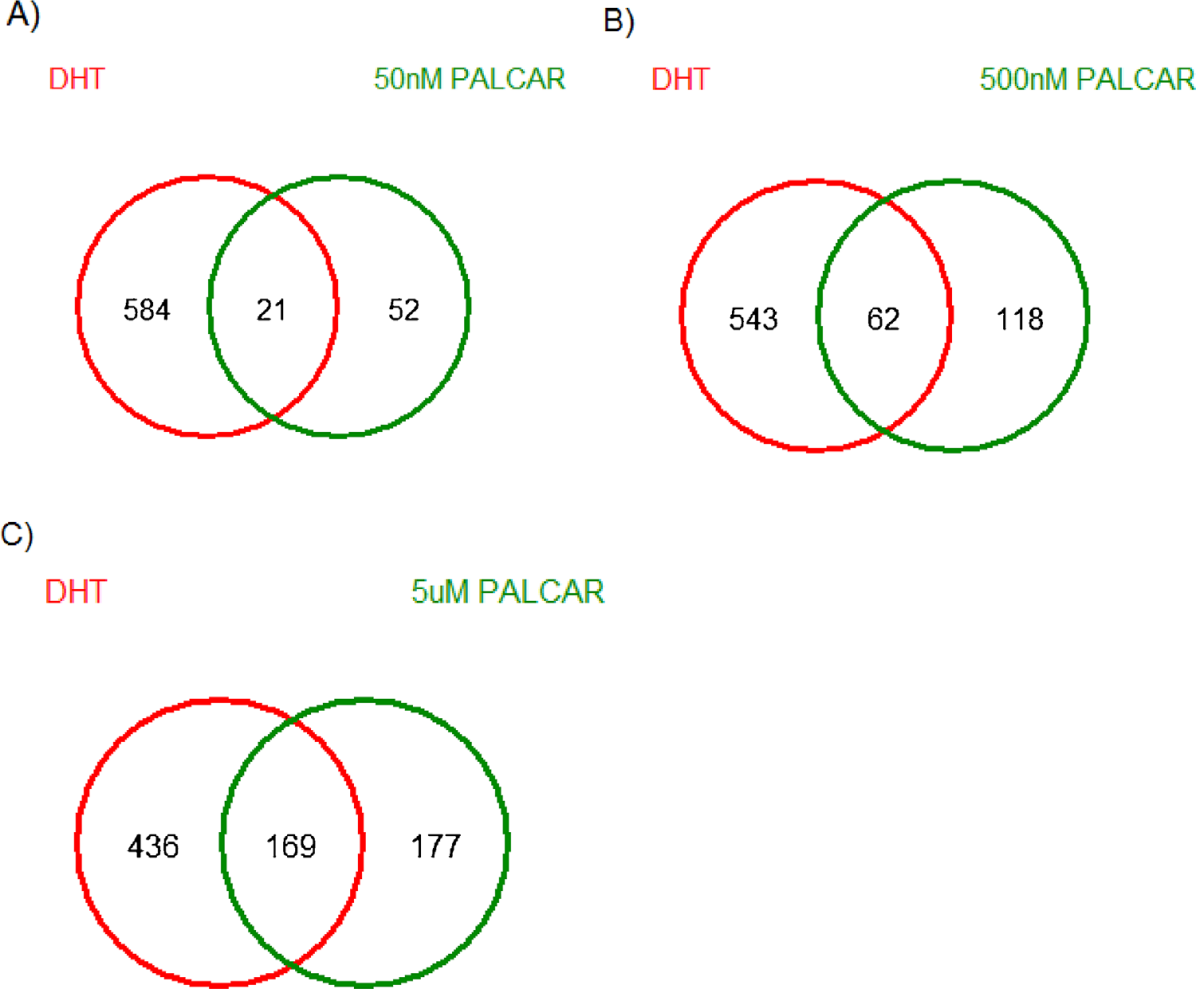
Venn diagram of genes common across palcar and DHT treatment in PNT1A cells. Cells were treated with palcar (0–5 μM) and 10 nM DHT for 8 hr. **A**, **B**, and **C** represent the genes common across 10 nM DHT and 50 nM, 500 nM, and 5 μM palcar, respectively.

Palcar effects on global gene expression were investigated by using Affymetrix Human Exon 1.0 ST arrays in non‐cancerous PNT1A prostate cells treated with increasing concentrations of palcar (0–5 μM) and DHT at the concentration of 10 nM. Palcar treatment was carried out using a range of concentrations similar to those found in human prostate tissue samples. The genes overlapped between each individual palcar concentration and DHT are illustrated in Fig. 6. There were a total of 21 (*P* < 0.05), 62 (*P* < 0.05), and 169 (*P* < 0.05) genes common between DHT and 50 nM, 500 nM, and 5 μM palcar, respectively. Functional analysis of the genes common between DHT and 5 μM palcar identified that these genes are involved in glycolysis/gluconeogenesis, cytokine‐cytokine receptor interaction, regulation of apoptosis, and cell death.

## DISCUSSION

Acylcarnitines are metabolites of fatty acid oxidation that accumulate as a result of mitochondrial dysfunctions. High levels of plasma acylcarnitines have been detected in chronic disorders, such as obesity and DM type 2, in which a metabolic disturbance has been observed 6, 36. Accumulation of acylcarnitines has also been observed in cancer 10, 11. A new pilot study reported a higher concentration of acylcarnitine in serum from patients with hepatocellular carcinoma compared with normal subjects 12. Another recent study revealed a significant difference of acylcarnitines levels between non‐small cell lung carcinoma patients and healthy controls by profiling their urine samples through LC‐MS 9. Higher plasma acylcarnitine levels have been detected in prostate cancer patients compared with BPH controls suggesting a potential role of these compounds as metabolic markers of the disease 13. However, to our knowledge, there are no previous reports of differences in tissue concentrations of acylcarnitines between prostate cancer patients and controls. This study aimed to investigate metabolic variations in terms of palcar levels between non‐cancerous and cancerous prostate tissue and its effects in in vitro prostate cell models. Palcar levels ranged from 0.02 μM to 0.1 μM with an average of 0.068 ± 0.03 μM in prostate cancer tissue. Lower levels of palcar (0.034 ± 0.02μM) were found in non‐cancerous tissue providing the first evidence of its accumulation in prostate cancer.

The difference observed between non‐cancerous and cancerous prostate tissue could be explained by a higher degree of lipid metabolism occurring as a result of the cancer reprogramming process. This might suggest mitochondrial overload of the long chain fatty acid (PA), which could be an indication of stimulated lipolysis that is a characteristic feature of cancer cells 37, 38. In vitro studies revealed that the uptake of PA by both malignant and benign cells was significantly higher than that of glucose 39, suggesting that fatty acid oxidation is a dominant energy source for the prostate cells. Mitochondrial overload with fatty acids may lead to the production of high levels of acetylCoA by β‐oxidation. It has been found that acetylCoA is a precursor for acetylcarnitine and ketone bodies 40, and it has been suggested that the formation of ketone bodies may inhibit or reduce the efficiency of the enzymes involved in TCA cycle. A less efficient TCA cycle would result in an insufficient integration with β‐oxidation, leading to incomplete fatty acid oxidation 36 and thus resulting in the accumulation of the intermediates of fatty acid oxidation, such as acylcarnitines, which would explain the accumulation of palcar in the prostate cancerous tissue observed in the current study. Another explanation would be a defect in carnitine palmitoyltransferase II (CPT‐II) enzyme involved in transferring the acyl group from acylcarnitines to the mitochondrial CoA. Nevertheless, further research is needed to investigate this hypothesis.

Palcar is an amphiphilic compound and is able to interact with both the phospholipid bilayer and the embedded proteins. This leads to a change in the microenvironment of the membrane that could affect the membrane bound proteins, which can be ion channels or receptors. The effect of palcar accumulation has been previously investigated 6, 11, 36 and has been associated with an alteration in Na^+^/K^+^ and Ca^2+^ pumps 27, 41, 42, 43. Previous studies have reported the effects of palcar in inducing Ca^2+^ influx 29 and pro‐inflammatory pathways 7 in human cell lines. To our knowledge, no other studies have reported the effects of palcar in prostate cells. The current study has explored the effects of palcar accumulation in terms of its action on the pro‐inflammatory cytokine IL‐6 and Ca^2+^ influx in prostate non‐cancerous (PNT1A and BPH‐1) and cancerous (DU145 and PC3) cells. Palcar induced IL‐6 secretion and gene expression in PC3 cells but a different effect was observed in PNT1A cells (Fig. 2) and androgen‐sensitive cancerous LnCaP cells (data not shown). We then compared the effect of palcar on IL‐6 secretion to those induced by an amphiphilic compound such as LPC. In contrast to palcar, high concentrations of LPC (50 and 100 μM) decreased the secretion of IL‐6 (Fig. 2C), which could be attributed to its action as a detergent in the presence of lipid membranes. PNT1A cell viability was not affected by palcar at any of the concentrations tested; however a significant decrease in cell viability has been observed by exposing cancerous PC3 cells to 50 μM and 100 μM of palcar. The different effect of palcar in cell survival remains unclear. It has been reported that PA dramatically reduced cell growth of neurons and intestinal muscle cells without affecting glia cell survival 44. PA‐induced cell death in vitro was explained by an increased production of palcar which resulted in higher levels of ROS. We could hypothesize that in our experimental conditions PNT1A cell survival was unchanged after palcar exposure in vitro because normal cells are less vulnerable to an increase of their basal levels of ROS compared to cancerous cells (PC3) 45. How this observation is relevant in vivo still requires further investigation.

In our study we also found a potential role of palcar in influencing Ca^2+^ cellular concentrations with interesting differences between cell types. Palcar at 50 μM was associated with Ca^2+^ influx in PC3 cells, but not in DU145, BPH‐1, or PNT1A cells (Fig. 4). The mechanism underlying palcar‐induced IL‐6 production via Ca^2+^ influx in PC3 cells has not been investigated. It is possible that the increase in [Ca^2+^]_i_ is important for the activation of transcription factors, such as NFκB, which is known to regulate IL‐6 secretion. Further studies are needed to investigate the downstream events of Ca^2+^‐induced signaling on IL‐6 secretion in PC3 cells, and to elucidate the mechanisms underlying the different response of prostate cells to palcar in terms of Ca^2+^ signaling.

Development and progression of prostate cancer has been widely associated with the androgen DHT, which activates androgen receptor (AR) signaling to promote prostate epithelial cell proliferation. IL‐6 has been shown to induce the activity of AR 46, 47, and therefore the effect of palcar in inducing IL‐6 in PC3 cells may suggest a potential role of this class of compounds in promoting cancer proliferation. Moreover, palcar effects on Ca^2+^ influx were similar to those induced by DHT (Fig. 5). DHT only induced Ca^2+^ influx in PC3 cells, but not in DU145, BPH‐1 or PNT1A cells, suggesting that palcar and DHT could act through similar mechanisms of action. The unresponsiveness of DU145, PNT1A, and BPH‐1 cells to both palcar and DHT may be related to an efficient Ca^2+^ buffer system in these cells that is not compromised by either palcar or DHT at the concentrations tested. Another explanation could be the presence of a cellular marker specifically modulated by both palcar and DHT that is only present in PC3 cells, but not in the other prostate cell lines.

To further investigate the similarity between palcar and DHT biological profiles we undertook a global analysis at the gene level in normal prostate PNT1A cells exposed to physiologically relevant palcar and DHT concentrations. The largest number of common genes (n = 169) was between 5 μM palcar and 10 nM DHT (*P* < 0.05) (Fig. 6). These were involved in cytokine signaling, apoptosis and glycolysis, which are important mediators of the action of DHT 48, 49, 50. These results provide preliminary evidence that palcar induces a DHT‐like response. DHT is a hormone required for prostate growth and development, and since palcar showed a DHT‐like activity, this suggests that palcar may have a role in prostate cancer progression.

In conclusion, these findings revealed a significant difference of palcar levels between non‐cancerous and cancerous prostate tissue, which highlight the potential use of palcar profiling as a biomarker for the metabolic disturbance associated with prostate cancer. High concentrations of palcar were associated with the induction of both IL‐6 and Ca^2+^ influx in vitro. The latter was also observed in response to DHT. Furthermore, global gene arrays showed that lower levels of palcar were associated with the induction of many changes in gene expression in the non‐cancerous prostate cells in common with DHT. Since DHT is a hormone associated with prostate growth, the DHT‐like effect of palcar may suggest a potential role of palcar in inducing prostate cancer progression.
